# Impact of FP2020 Program on High-Risk Pregnancies and Under-Five Mortality in the Democratic Republic of the Congo: Evidence from a Quasi-Experimental Analysis Using Demographic and Health Surveys (2013–2023)

**DOI:** 10.3390/healthcare14040521

**Published:** 2026-02-18

**Authors:** Yves M. Kashiya, Pierre Z. Akilimali, Paul-Samsom D. Lusamba, Yves Coppieters

**Affiliations:** 1 Department of Biostatistics and Epidemiology, School of Public Health, University of Kinshasa, Kinshasa P.O. Box 11850, Democratic Republic of the Congo; 2 Patrick Kayembe Research Center, Kinshasa School of Public Health, University of Kinshasa, Kinshasa P.O. Box 11850, Democratic Republic of the Congo; pierre.akilimali@unikin.ac.cd; 3 Department of Nutrition, School of Public Health, University of Kinshasa, Kinshasa P.O. Box 11850, Democratic Republic of the Congo; 4 Department of Biostatistics and Epidemiology, School of Public Health, Université Libre de Bruxelles (ULB), Erasme, 1070 Brussels, Belgium

**Keywords:** family planning, modern contraceptives, maternal health, child mortality, Difference-in-Differences, FP2020, Democratic Republic of the Congo, impact evaluation

## Abstract

Background: Family planning remains a cornerstone of reproductive health strategies to reduce maternal and child mortality by preventing unintended and high-risk pregnancies. Despite the implementation of the FP2020 initiative, empirical evidence on its population-level impact in the Democratic Republic of the Congo (DRC) remains scarce. This study examined the association between modern contraceptive use as a proxy for exposure to the FP2020 era expansion of family planning services and high-risk pregnancies and under-five mortality during the FP2020 in the DRC era. Methods: A quasi-experimental Difference-in-Differences (DiD) design was applied using Demographic and Health Survey (DHS) data from 2013 (pre-intervention) and 2023 (post-intervention). Women aged 15–49 years with at least one live birth were included for maternal outcomes, while all live-born children within five years preceding each survey were analyzed for child outcomes. Weighted analyses employed Linear Probability Models (LPMs), adjusting by potential confounder variables. Results: The prevalence of high-risk pregnancies among modern contraceptive users declined from 58.6% in 2013 to 54.5% in 2023, while under-five mortality decreased from 10.4% to 5.9% over the same period. DiD estimates revealed a significant reduction in high-risk pregnancies among users in urban areas (β = −0.067) (95% CI: −0.133 to −0.003), and a substantial decline in under-five mortality in rural areas (β = −0.031) (95% CI: −0.059 to −0.002). Results remained robust across model specifications, and a parallel trends test confirmed model validity (*p* > 0.5). Conclusions: Findings demonstrate that the FP2020 initiative and increased modern contraceptive use contributed to measurable reductions in maternal and child health risks in the DRC. Expanding access to family planning within universal health coverage (UHC) frameworks could further reduce health inequalities and accelerate progress toward the Sustainable Development Goals (SDGs).

## 1. Introduction

Family planning (FP) is a key public health intervention that contributes to the reduction of unintended pregnancies, maternal and child mortality, and to broader development outcomes such as women’s empowerment, education, and poverty reduction [1]. The use of modern contraceptive methods is estimated to prevent approximately 30% of maternal deaths and 10% of child deaths globally [2,3,4]. By ensuring optimal birth spacing and reducing high-risk pregnancies, FP plays a crucial role in improving reproductive and child health outcomes [5,6].

Globally, the modern contraceptive prevalence rate (mCPR) among married women of reproductive age increased modestly from 55.0% in 2000 to 57.1% in 2019 [7]. In low-income countries, however, uptake remained limited. To address this gap, the FP2020 initiative was launched in 2012, encouraging African countries to commit additional financial and policy resources to FP services. As a result, the mCPR in Sub-Saharan Africa increased from 18.5% in 2010 to 24.4% in 2020 [8]. Sixteen African countries had reached the acceleration phase of contraceptive uptake (“stage 2” of the S-curve) by 2022 [9]. The FP2030 agenda has since taken over, setting more ambitious goals to achieve universal access to reproductive health.

While many African countries have engaged in impact evaluations of health policies, few have specifically assessed the direct contribution of FP to maternal and child health outcomes [10,11,12]. Yet, several studies suggest that improved access to FP, particularly postpartum, can significantly increase the uptake of long-acting reversible methods such as implants and enhance birth spacing [13]. A multi-country analysis of 205 Demographic and Health Surveys (DHS) between 1985 and 2013 showed that increased contraceptive use is associated with declines in high-risk pregnancies (short intervals, high parity, advanced maternal age) [14]. In much of Sub-Saharan Africa, FP is predominantly used to space rather than limit births, contributing to the region’s persistently high fertility rates [15,16].

The Democratic Republic of the Congo (DRC) continues to face significant reproductive health challenges. According to DHS data, modern contraceptive prevalence among women aged 15–49 increased modestly from 6% in 2007 to 8.1% in 2013. In urban areas, it rose from 10% to 15%, while rural coverage remained extremely low (5% in 2013). These low rates coexist with alarming health indicators: a fertility rate of 6.6 children per woman, maternal mortality of 846 deaths per 100,000 live births, and child mortality of 104 per 1000 live births [17].

To reverse these trends, the DRC joined FP2020 in 2013 and launched its first National Strategic Plan for FP (2014–2020). Investments were made in contraceptive procurement and supply, and technical coordination was reinforced through the establishment of multisectoral technical committees (CTMP) at national and provincial levels [8,18,19]. In 2018, the mCPR reached 16.1% (MICS 2017–2018), though regional and age-group disparities remained [20]. The country’s second strategic plan (2021–2025) aligns with the FP2030 targets, aiming to raise mCPR to 30%.

Despite policy progress, no national study has rigorously evaluated maternal and child health outcomes associated with modern contraceptive use during the FP2020 era in the DRC. Such evidence is critical to inform decisions on how best to integrate FP into the country’s broader universal health coverage (UHC) agenda [21]. This study assessed the impact of the FP2020 program use on self-reported high-risk pregnancies and child mortality in the DRC, using DHS data from 2013 and 2023. High-risk pregnancy was selected as the maternal health indicator instead of maternal mortality due to data limitations inherent to population-based surveys.

## 2. Materials and Methods

### 2.1. Study Design

This study employs a quasi-experimental design based on the Difference-in-Differences (DiD) approach, using secondary data from the Demographic and Health Surveys (DHS). The FP2020 initiative was launched in the Democratic Republic of the Congo in 2014. In line with this timeline, the 2013 Demographic and Health Survey (DHS) was designated as the pre-intervention baseline, while the 2023 DHS represented the post-intervention period.

Specifically, the analysis focuses on the prevalence of self-reported high-risk pregnancies and under-five child mortality. Although this is not a randomized controlled experiment, the availability of two survey waves combined with the presence of an exposed group (modern contraceptive users) and an unexposed group (non-users) allows for the construction of a counterfactual scenario through a robust impact evaluation method [21,22].

### 2.2. Data Sources

We used nationally representative data from the DRC DHS conducted in 2013–2014 and the most recent DHS conducted in 2023–2024. The DHSs use a multistage stratified sampling design and collect standardized information on fertility, contraceptive use, maternal and child health, and household characteristics. These datasets are comparable over time and provide rich variables suitable for longitudinal policy evaluation in repeated cross-sectional formats.

### 2.3. Study Population and Sample

This study focuses on two sub-populations, defined according to the health indicators under investigation:

- For maternal health outcomes, the sample includes women aged 15 to 49 years who have had at least one live birth, regardless of the time elapsed since the delivery, and who responded to both the family planning module and the section on high-risk pregnancies.
- For child health outcomes, the analysis includes all children born to these women during the same reference period.

Observations with missing data on contraceptive use, reported high-risk pregnancies, or child survival status were excluded to ensure consistency and robustness of the analysis.

### 2.4. Intervention

In the DRC, the FP2020 initiative was implemented through successive National Family Planning Strategic Plans (2014–2020 and 2021–2025). The intervention focused on strengthening contraceptive supply chains, building provider capacity, particularly for long-acting methods, promoting community-based distribution and media-driven demand generation, and establishing multisectoral coordination bodies (CTMP). It also mobilized domestic and external funding to reduce user costs and integrated family planning into the essential health services package under the universal health coverage framework. In this study, the implementation of FP2020 in the Democratic Republic of the Congo is leveraged as an exogenous policy intervention. Given its national scale and focus on improving access to modern contraceptive methods, FP2020 provides an appropriate context to assess the causal effects of family planning initiatives on maternal and child health outcomes [19,23].

### 2.5. Variables

- Use of modern contraceptive methods (treatment): The main exposure variable is defined as the current use of any modern contraceptive method at the time of the survey. Modern methods include implants, intrauterine devices (IUDs), pills, injectables, and condoms. A binary variable was created (1 = user of a modern method, 0 = non-user). Individual-level modern contraceptive use is employed as a proxy indicator of exposure to the FP2020 era expansion of family planning services. This approach assumes that FP2020 increased the availability, affordability, and acceptability of modern contraceptive methods, thereby influencing individual uptake. It reflects longer-term behavioral adoption rather than a point-in-time intervention.
- Maternal indicator (outcome variable 1): High-risk pregnancy is defined as the self-report by a woman of having experienced at least one obstetric risk factor during her most recent pregnancy. Risk factors include extreme maternal age (women younger than 18 years or older than 34 years), high parity (number of live births of five or more), and short birth intervals (as less than 24 months between the two most recent consecutive live births), conditions identified by the WHO [16]. A binary variable was constructed (1 = high-risk pregnancy, 0 = no identified risk). To construct the high-risk pregnancy variable, women without any live births (nulliparous) were excluded from the analysis, while no restriction was applied regarding the timing of the last birth. Maternal age at the last delivery was computed irrespective of the time elapsed since the delivery, to ensure broader inclusion.
- Child health indicator (outcome variable 2): Under-five mortality is defined as the death of a child before reaching the age of 60 months. A binary variable was constructed, coded as 1 if the child died before age five, and 0 if the child was alive at the time of the survey or had survived beyond five years. Under-five mortality was retained due to its higher frequency and established comparability across DHS rounds. We included all deaths occurring before age five among children observed during the study period, to fully capture under-five mortality risk.
- Post-intervention: A binary variable was used to indicate the timing of data collection relative to the FP2020 initiative. The variable was coded as 1 for the year 2023, corresponding to the post-intervention period following the implementation of FP2020, and 0 for the year 2013, representing the pre-intervention period before the program’s adoption.
- Control variables (Table 1)

### 2.6. Statistical Analysis

Descriptive analyses were conducted to characterize the study population and to examine the distribution of key explanatory variables and maternal and child health outcomes. Sociodemographic characteristics of women aged 15 to 49 who had at least one live birth in each survey (2013 and 2023) were described by survey year. Frequencies and percentages were calculated, and statistical tests were used to assess differences between groups and survey periods (Chi-square tests for categorical variables).

The prevalence of reported high-risk pregnancies and under-five mortality rates was also estimated and compared by contraceptive use and survey year. Visualizations were generated to show trends in these outcomes between 2013 and 2023 by modern contraceptive use status.

To describe the prevalence of high-risk pregnancies, only the merged Individual Recode (IR) datasets from the 2013 and 2023 DHSs were used. Using the IR files exclusively avoids the duplication of women that occurs when merging with the Birth Recode (BR) datasets, ensuring accurate counts for descriptive analyses and preventing bias related to the number of children per woman.

Additionally, a parallel trends analysis was conducted using data from the 2007 and 2013 DHSs to test the key assumption of the Difference-in-Differences (DiD) model, namely that the pre-intervention outcome trajectories were similar across treatment and comparison groups. This step was essential to validate the DiD estimation strategy. Prior to multivariate modelling, a correlation matrix (or Variance Inflation Factors) was computed to assess multicollinearity among covariates, ensuring model validity. All VIF values were below conventional thresholds (VIF < 5), indicating no evidence of problematic multicollinearity.

The main multivariate analysis relied on the Linear Probability Model (LPM), selected for its straightforward interpretation of probability differences in binary outcomes and its common use in DiD frameworks. In this context, the LPM allows interaction between users and the post-intervention period to be directly interpreted as an absolute percentage point change, facilitating policy-relevant interpretation. The model estimates the differential change over time between users and non-users of contraception, capturing the net association observed during the FP2020 policy period rather than predicting individual-level outcome probabilities. To account for clustering at the primary sampling unit (PSU) level and ensure valid inference, analyses were weighted using DHS sampling weights and robust standard errors were applied.

The DiD regression model was specified as follows:

*Yit* = *β*_0_ + *β*_1_ Post*_t_* + *β*_2_ Treatment*_i_* + *λ*(Post*_t_* × Treatment*_i_*) + X*_it_* + *ϵ_it_*

where *Yit* is the binary outcome variable for individual i at time t (high-risk pregnancy or under-five mortality); Post*_t_* is a time dummy variable (1 if 2023, post-FP2020 intervention; 0 if 2013, pre-intervention); Treatment*_i_* indicates modern contraceptive use (1 = user, 0 = non-user); *λ* is the DiD estimator capturing the net intervention effect (effect of the FP2020 program in the DRC); X*_it_* is a vector of covariates; and *ϵ_it_* is the error term.

Subgroup analyses stratified by place of residence (urban vs. rural), educational attainment, and wealth quintile were also conducted to explore potential heterogeneity in the intervention effect.

### 2.7. Ethical Considerations

This study is based on the analysis of secondary data obtained from Demographic and Health Surveys (DHSs), which are fully anonymized and publicly accessible through the DHS Program. The original data collection was authorized by the relevant national health authorities in the Democratic Republic of the Congo and carried out in collaboration with ICF International, in accordance with international ethical standards.

In line with the policies of the Kinshasa School of Public Health Committee regarding the use of publicly available and anonymized data, this analysis did not require additional ethical approval. No personally identifiable information was used, ensuring full compliance with confidentiality and participating protection principles.

## 3. Results

### 3.1. Impact of Modern Contraceptive Prevalence Rate on Maternal Health Outcomes

**Figure 1:**
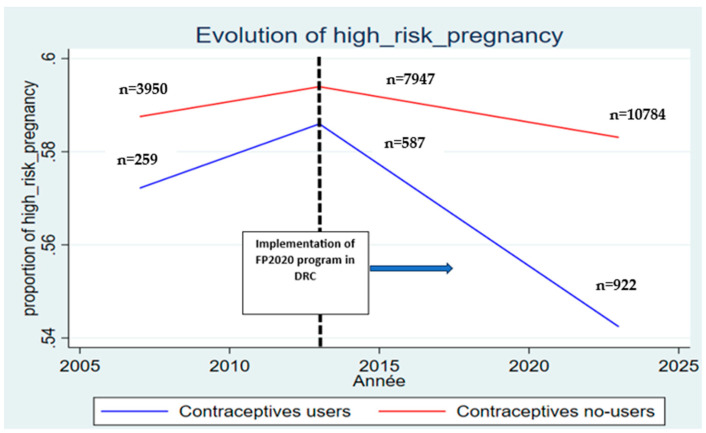
Trend of High-Risk Pregnancy Between Contraceptive Users And Non-Users from 2007 to 2023.

The descriptive analysis provided substantial insights into the distribution of high-risk pregnancies and under-five mortality across the study periods, stratified by maternal use of modern contraceptives. In 2013, 59.4% of non-users and 58.6% of users reported experiencing at least one obstetric risk factor during their most recent pregnancy. A decade later, by 2023, the prevalence of high-risk pregnancies had decreased to 58.2% among non-users and to 54.5% among users of modern contraception (Table 2). This decline was more pronounced among women who adopted modern contraceptive methods, suggesting a potential protective effect over time. The parallel trends test, conducted over the pre-intervention period (2007–2013), shows that the coefficients for the pre-treatment years are not statistically different from zero (*p* = 0.84). (Figure 1).

Examining the sociodemographic characteristics revealed that women who used modern contraceptive methods were more likely to be educated, reside in urban areas, and belong to the higher quintiles of the household wealth index. In 2013, 60.6% of modern contraceptive users resided in urban areas compared to 31.4% of non-users. By 2023, the proportion of urban residents among users decreased slightly to 54.0%, while non-users remained predominantly rural at 67.3%. The age distribution differed moderately between groups. Across periods, users were consistently more likely to have been exposed to media and to have visited a health facility in the preceding 12 months. Employment status also differed between groups, with users being more frequently engaged in paid work, particularly in 2023.

Education levels also differed markedly: a greater proportion of contraceptives users had attained secondary or higher education compared to non-users across both periods (Table 2).

This Difference-in-Differences (DiD) approach was employed to evaluate the impact of modern contraceptive use on the probability of experiencing a high-risk pregnancy, stratified by place of residence. In urban areas, the analysis using the Linear Probability Model indicated a significant reduction in the probability of high-risk pregnancy among women using modern contraceptive methods. Specifically, the interaction term between contraceptive use and the post-intervention period yielded a coefficient of −0.067 (95% CI: −0.133 to −0.003), suggesting a 6.7 percentage point decrease in the probability of high-risk pregnancy among users after 2023.

In rural areas, however, the results from LPM [II] did not reveal a statistically significant effect. The coefficient was −0.005 (95% CI: −0.088 to 0.093), indicating no meaningful change in high-risk pregnancy prevalence among rural women associated with contraceptive use. When the analysis was extended to the total sample using LPM [III], the DiD coefficient was −0.046 (95% CI: −0.099 to 0.006), which also failed to reach statistical significance (Table 3).

Beyond the primary exposure, several covariates demonstrated consistent associations across models. Increasing maternal age was positively correlated with the likelihood of high-risk pregnancy, underscoring the elevated obstetric risks associated with advancing maternal age. Educational attainment, particularly secondary and higher education, was inversely related to high-risk pregnancies, suggesting a protective effect of education. Similarly, women belonging to the richest wealth quintile exhibited a lower probability of high-risk pregnancies compared to their poorer counterparts.

### 3.2. Impact of Modern Contraceptive Prevalence Rate on Child Health Outcomes

**Figure 2:**
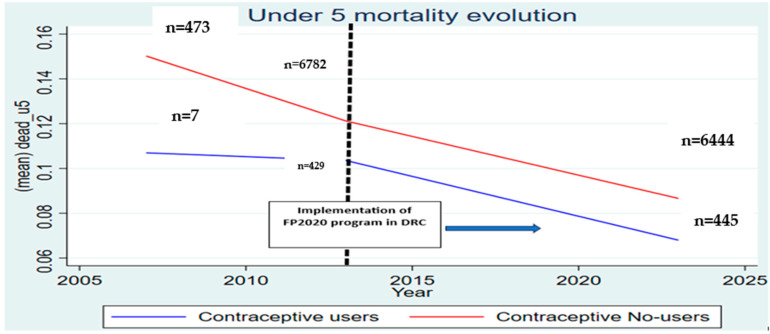
Trend of Under-Five Mortalities Between Contraceptive Users And Non-Users from 2007 to 2023.

Turning to under-five mortality, the descriptive statistics showed a notable decline over the decade. In 2013, 12.1% of children born to women in the sample died before reaching five years of age. Among non-users of modern contraception, the under-five mortality rate stood at 12.2%, while among users, it was slightly lower at 10.4%. By 2023, the situation had improved significantly: under-five mortality declined to 8.3% among children of non-users and further to 5.9% among children of users (Table 4). The overall under-five mortality rate for the full sample fell to 8.0% in 2023, confirming steady progress in child survival. The parallel trends test confirms the absence of significant divergence between the treatment and control groups prior to the program (*p =* 0.57), supporting the validity of the identification strategy (Figure 2).

Sociodemographic characteristics associated with under-five mortality mirrored those observed for high-risk pregnancies. In both years, users were likely to be multiparous women aged 35 years or older (Table 4).

The Difference-in-Differences (DiD) analysis was applied to assess the impact of modern contraceptive use on under-five mortality, stratified by place of residence. In urban areas, results from the Linear Probability Model indicated a non-significant interaction term between modern contraceptive use and the post-intervention period, with a coefficient of 0.015 (95% CI: −0.005 to 0.033). This suggests no substantial reduction in under-five mortality attributable to contraceptive use in urban settings.

In rural areas, the model revealed a statistically significant association. The interaction coefficient was −0.031 (95% CI: −0.059 to −0.002), indicating a 3.1 percentage point reduction in the probability of under-five mortality among users compared to non-users post-2023. When considering the entire sample, the interaction coefficient was −0.010 (95% CI: −0.026 to 0.007), a result that did not reach statistical significance (Table 5).

Other covariates displayed consistent effects across the models. A female child was associated with a decrease risk of under-five mortality. Higher maternal parity (five children or more) was strongly associated with an increased risk of under-five mortality.

Socioeconomic factors also played a substantial role. Belonging to richer or richest households was associated with a significantly lower risk of under-five mortality compared to poorer households.

## 4. Discussion

This study employed a quasi-experimental Difference-in-Differences (DiD) approach to assess the impact of modern contraceptive use during the FP2020 program on the prevalence of high-risk pregnancies and under-five mortality in the Democratic Republic of the Congo (DRC) between 2013 and 2023. Utilizing nationally representative Demographic and Health Survey (DHS) data at two different time points strengthened the internal validity of the study. Notably, the parallel trends assumption was verified using the 2007 DHS data, confirming the validity of applying DiD analysis. This design effectively controls for time-invariant confounding factors.

Descriptive analyses highlighted a gradual reduction in the prevalence of high-risk pregnancies and under-five mortality over the study period. Among women using modern contraceptive methods, the prevalence of high-risk pregnancies decreased from 58.6% in 2013 to 54.5% in 2023, whereas the decline was more modest among non-users (from 59.4% to 58.2%). Similarly, under-five mortality among children of mothers using modern contraceptives fell from 10.4% to 5.9%, compared to a decrease from 12.2% to 8.3% among non-users, indicating a more substantial improvement among users.

The DiD analysis confirmed these trends. For high-risk pregnancies, the interaction between modern contraceptive use and the post-intervention period revealed a statistically significant reduction among urban residents. Higher access to services (availability, trained providers, long-acting methods, and continuity of care) can more directly result in a reduction of high-risk pregnancies through better planning and spacing

However, a significant decrease in under-five child mortality was observed in rural areas where the baseline risk of mortality is often higher; even a modest improvement in birth spacing and in limiting closely spaced pregnancies can produce a more noticeable relative effect on child survival. This finding suggests that the use of modern contraceptives contributed to improved child survival rates in rural settings, where baseline risks tend to be higher.

Our findings align with global evidence emphasizing that universal access to modern contraceptives not only reduces high-risk pregnancies but also plays a crucial role in decreasing maternal and newborn mortality, underscoring the necessity of integrating family planning services into universal health coverage initiatives [24,25].

When compared to existing literature, our findings are consistent with several studies. Adenini al. (2015) in Nigeria, Ankita Shukla (2020) in India and several other authors found a significant association between modern contraceptive use and reduced under-five mortality [16,25,26,27]. Similarly, Bawuah et al. (2025) reported that expanding access to family planning services in sub-Saharan Africa had a notable effect on birth spacing and reductions in maternal and child health risks [21,26]. Our findings also align with the work of Cleland et al. (2012) and Bongaarts (2014), who demonstrated that improved access to reproductive health services can reduce high-risk births, a key driver of under-five mortality [28,29].

Jahanfar et al. (2024) found a 30% reduction in the risk of high-risk pregnancies among contraceptive users compared to non-users, reinforcing the positive maternal health impacts of modern contraceptive use [26]. Similarly, Adedini et al. (2015) highlighted that an unmet need for family planning was significantly associated with higher under-five mortality, emphasizing the crucial role of contraceptive access in improving child survival outcomes [25].

Jahanfar et al. (2024) documented significant reductions in the risk of obstetric complications linked to modern contraceptive use, findings consistent with the decrease we observed in urban areas of the DRC [26]. Other authors also demonstrated that higher maternal education and wealth significantly improved modern contraceptive use. This may be explained by their better understanding of the importance of family planning and their greater ability to access healthcare services, even when faced with direct out-of-pocket payments [6,16,30,31].

Exposure to media has been identified as a key driver in increasing the intention to use modern contraceptive methods among women in sub-Saharan Africa [21]. Although media exposure differed between users and non-users in our findings, it was not significantly associated with high-risk pregnancy after multivariable adjustment, indicating that observed differences may reflect correlated socioeconomic characteristics.

Interestingly, while our findings indicated a more significant impact on under-five mortalities in rural areas, other studies, such as Benova et al. (2019), reported more pronounced benefits in urban settings, likely due to better healthcare service availability [32]. However, recent efforts in the DRC to extend reproductive health services to rural areas under the FP2020 initiative may explain the stronger impact observed in rural populations in our study.

### Limitations of Study

Nonetheless, this study has some limitations. The primary limitation is the use of cross-sectional survey data, which, despite adjustments with DiD, cannot fully eliminate residual confounding and establish strict causal inference. In addition, FP2020 was implemented as a national policy, but the intensity and timing of activities likely varied across provinces and over time due to differences in partner presence, funding flows, health system capacity, and demand-generation efforts. Because DHS data do not capture subnational variation in program implementation, this geographic and temporal heterogeneity could not be explicitly measured and may have contributed to heterogeneity in the observed associations.

Contextual variables such as distance to healthcare facilities and quality of services were not available, limiting deeper exploration of mediating factors. Moreover, our analysis focused on current contraceptive use and did not capture changes in use over time, which could influence the outcomes. Contraceptive use was self-reported and measured at the time of survey, which may not perfectly align temporally with pregnancy or child health outcomes. Restricting analyses to narrowly defined recent birth windows improves temporal alignment but does not fully address potential exposure misclassification or reverse causality and substantially reduces sample size and statistical power.

Future research should consider longitudinal designs to better understand the dynamic effects of contraceptive use on maternal and child health outcomes. Mixed methods approaches that combine quantitative and qualitative analyses would also provide a more comprehensive understanding of the barriers to contraceptive use, especially in rural settings. Additionally, expanding the scope to examine other outcomes, such as maternal morbidity and early childhood development, could offer further insights into the broader impacts of family planning interventions.

## 5. Conclusions

This study provides evidence of a statistically significant association between modern contraceptive use in a specific subpopulation with high-risk pregnancies and under-five mortality in the Democratic Republic of the Congo, addressing critical maternal and child health challenges. Using a rigorous Difference-in-Differences approach, we demonstrated that family planning interventions, particularly those linked to the FP2020 initiative, have improved reproductive health outcomes, especially in rural areas for child survival and urban settings for maternal health risks. These findings underscore the need for expanded access to modern contraception, combined with strategies that ensure financial protection for vulnerable populations. Integrating family planning services into universal health coverage schemes can reduce out-of-pocket expenditures and enhance equitable access.

Policymakers should strengthen investments in reproductive health, expand subsidized services, and promote community-based education programs. Ensuring that financial barriers are minimized will be crucial to sustaining progress and accelerating the achievement of Sustainable Development Goals related to maternal and child health.

## Figures and Tables

**Table 1:** Control variables use for outcome 1,2.

Variable	Operational Definition	Measurement Modalities	Used for
Maternal age	Mother’s age at the survey	Continuous (Years)	Outcome 1,2
Parity	Number of live births reported by the mother	Binary (<5 children or ≥5 children)	Outcome 2
Child’s sex	Biological sex of the child	Binary (Male or Female)	Outcome 2
Mother’s education level	Mother’s highest level of education attained	Categorical (None, Primary, Secondary, Higher)	Outcome 1,2
Religion	Beliefs of the mother	Categorical (Christianism, Islam, Animism, No religion, Others)	Outcome 1,2
Household wealth index	Economic status based on household asset ownership, divided into quintiles	Categorical (Poorest, Poorer, Middle, Richer, Richest)	Outcome 1,2
Media exposure	Access to mass media (newspapers, radio, or television)	Binary (Exposed or Not exposed)	Outcome 1
Employment status	Whether the mother is currently employed	Binary (Employed or Not employed)	Outcome 1
Visited health facility in the last 12 months	Visited the health facility	Binary (Yes or No)	Outcome 1
Health aid	To have skilled assistance	Binary (Yes or No)	Outcome 2
Place of residence	Type of residence	Binary (Urban or Rural)	Outcome 1,2

**Table 2:** Descriptive characteristics of women by modern contraceptive use, 2013 vs. 2023.

Sample Characteristics	Use of Modern Contraceptive Method in 2013			Use of Modern Contraceptive Method in 2023		
	Yes	No	Total	Yes	No	Total
	*n* (%)	*n* (%)	*n* (%)	*n* (%)	*n* (%)	*n* (%)
High-risk pregnancy	1004	13,178	14,182	1636	17,920	19,556
No risk	417 (41.1)	5231 (40.6)	5648 (40.7)	714 (45.5)	7136 (41.8)	7850 (42.2)
At risk	587 (58.6)	7947 (59.4)	8534 (59.3)	922 (54.5) *	10,784 (58.2)	11,706 (57.8)
Age categories (year)						
15–24	257 (26.4)	3313 (24.7)	3570 (24.8)	362 (21.2)	4684 (24.3)	5046 (24.0)
25–34	419 (43.7)	5239 (40.7)	5658 (41.0)	659 (40.6)	6252 (36.0)	6911 (36.5)
35+	328 (30.0)	4626 (34.7)	4954 (34.3)	615 (38.1) *	6984 (39.7)	7599 (39.5)
Highest educational level						
No education	100 (9.5)	2903 (19.3)	3003 (18.5)	169 (9.6)	4098 (19.9)	4267 (18.8)
Primary	269 (26.3)	5724 (42.3)	5993 (40.9)	369 (19.2)	5956 (28.7)	6325 (27.7)
Secondary	585 (59.2)	4365 (36.6)	4950 (38.5)	986 (61.6)	7468 (47.2)	8454 (48.7)
Higher	50 (5.0) ***	186 (17.6)	236 (2.0)	112 (9.6)	398 (4.2)	510 (4.8)
Wealth index						
Poorest	98 (8.5)	3486 (21.9)	3584 (20.7)	229 (9.4)	5175 (22.2)	5404 (20.8)
Poorer	128 (12.3)	2900 (21.4)	3028 (20.7)	273 (13.2)	4641 (21.4)	4914 (20.5)
Middle	140 (11.7)	2721 (20.6)	2861 (19.8)	323 (15.6)	3884 (20.3)	4207 (19.8)
Richer	251 (26.9)	2258 (18.2)	2509 (19.0)	434 (27.0)	2707 (19.1)	3141 (20.0)
Richest	387 (40.7) ***	1813 (17.9)	2200 (19.8)	377 (34.8) ***	1513 (17.0)	1890 (18.9)
Religion						
Christianism	963 (96.4)	12,575 (96.2)	13,538 (96.2)	929 (53.8)	10,674 (57.1)	11,603 (56.7)
Islam	19 (1.7)	213 (1.3)	232 (1.4)	644 (42.9)	6291 (38.3)	6935 (38.8)
Animist	4 (0.5)	56 (0.4)	60 (0.4)	22 (1.1)	224 (1.3)	246 (1.3)
No religion	5 (0.8)	139 (1.0)	144 (1.0)	24 (2.2)	501 (3.3)	525 (3.2)
Others	9 (0.6)	161 (1.1)	170 (1.0)	0 (0.0)	19 (0.0)	19 (0.0)
Visited health facility in the last 12 m						
No	527 (49.4)	7527 (55.6)	8054 (55.1)	951 (58.8)	11,658 (64.4)	12,609 (63.8)
Yes	477 (50.6) *	5646 (44.4)	6123 (44.9)	685 (41.2)	6262 (35.6)	6947 (36.2)
Media exposure						
Not exposed	776 (74.1)	12,025 (89.2)	12,801 (87.9)	1108 (64.2)	14,857 (79.8)	15,965 (78.1)
Exposed	228 (25.9) ***	1153 (10.8)	1381 (12.1)	528 (35.8) ***	3063 (20.2)	3591 (21.9)
Currently working						
No	266 (27.6)	3032 (23.5)	3298 (23.8)	506 (33.13	6570 (38.2)	7076 (37.6)
Yes	737 (72.4) *	10,110 (76.5)	10,847 (76.2)	1130 (66.7) **	11,350 (61.8)	12,480 (62.4)
Type of residence						
Urban	624 (60.6) ***	3956 (31.4)	4580 (33.9)	812 (54.0)	4807 (32.7)	5619 (35.0)
Rural	380 (39.4)	9222 (68.6)	9602 (66.1)	824 (46.0) ***	13,113 (67.3)	13,937 (65.0)

Legend: (1) * < 0.05; (2) ** < 0.001; (3) *** < 0.0001.

**Table 3:** Difference-in-Differences estimates of the effect of modern contraceptive use on the probability of high-risk pregnancy, stratified by place of residence.

Variables	LPM [I]		LPM [II]		LPM [III]	
	β	IC95%	β	IC95%	β	IC95%
Treatment x 2023	−0.067 *	[−0.133; −0.003]	0.005	[−0.088; 0.093]	−0.046	[−0.099; 0.006]
Year (2023)	−0.055 **	[−0.089; −0.021]	0.007	[−0.013; 0.028]	−0.013	[−0.030; 0.004]
Treatment	0.05	[−0.000; 0.095]	0.034	[−0.040; 0.107]	0.051 *	[−0.009; 0.093]
Age (year)	0.02 ***	[0.018; 0.02]	0.02 ***	[0.019; 0.021]	0.02 ***	[0.019; 0.021]
Highest educational level						
Primary	−0.0002	[−0.051; 0.05]	−0.0033	[−0.023; 0.0171]	−0.002	[−0.016; 0.021]
Secondary	−0.111 ***	[−0.160; −0.061]	−0.077 ***	[−0.101; −0.052]	−0.084 ***	[−0.101; −0.059]
Higher	−0.24 ***	[−0.304; −0.168]	−0.289 **	[−0.486; −0.093]	−0.228 ***	[−0.273; −0.172]
Wealth index						
Poorer	−0.032	[−0.097; 0.033]	−0.001	[−0.022; 0.020]	−0.003	[−0.023; 0.017]
Middle	−0.013	[−0.066; 0.040]	0.016	[−0.007; 0.039]	0.017	[−0.004; 0.037]
Richer	−0.035	[−0.081; 0.011]	0.037 *	[0.003; 0.068]	0.024	[−0.002; 0.050]
Richest	−0.099 **	[−0.147; −0.051]	0.152 *	[0.02; 0.280]	−0.04 *	[−0.072; −0.008]
Media exposure						
Exposed	−0.005	[−0.023; 0.033]	−0.012	[−0.045; 0.019]	−0.018	[−0.023; 0.019]
Employed						
Yes	−0.019	[−0.045; 0.080]	0.010	[−0.011; 0.031]	−0.00001	[−0.016; 0.016]
Type of residence						
Rural	-	-	-	-	0.030 *	[0.075;0.053]
Observations	10,133		23,297		33,430	
Stratification	Urban		Rural		All	

Legend: (1) * < 0.05; (2) ** < 0.001; (3) *** < 0.0001.

**Table 4:** Descriptive characteristics of child by mother’s modern contraceptive use, 2013 vs. 2023.

Sample Characteristics	Use of Modern Contraceptive Method in 2013			Use of Modern Contraceptive Method in 2023		
	Yes	No	Total	Yes	No	Total
	*n* (%)	*n* (%)	*n* (%)	*n* (%)	*n* (%)	*n* (%)
Under-five mortalities	4021	54,310	58,331	6508	73,434	79,942
Child alive or dead after 5 years	3592 (89.6)	47,528 (87.8)	51,120 (87.9)	6063 (94.1)	66,990 (91.7)	73,053 (92.0)
Child dead before 5 years	429 (10.4) *	6782 (12.2)	7211 (12.1)	445 (5.9) ***	6444 (8.3)	6889 (8.0)
Child sex						
Male	2093 (51.6)	27,741 (50.3)	29,834 (50.4)	3330 (50.6)	37,753 (51.1)	41,083 (51.0)
Female	1972 (48.4)	27,470 (49.7)	29,442 (49.6)	3240 (49.4)	36,567 (48.9)	39,807 (48.9)
Age of mother (year)						
15–24	438 (11.4)	5801 (10.4)	6239 (10.4)	625 (9.5)	8649 (10.8)	9274 (10.7)
25–34	1511 (39.8)	20,471 (37.8)	21,982 (38.0)	2285 (35.2)	23,965 (32.8)	26,250 (33.0)
35+	2166 (48.8)	28,939 (51.8)	31,055 (51.6)	3660 (53.7)	41,706 (56.4)	45,366 (56.3)
Health aid						
Unskilled/No assistance	143 (9.6)	4720 (21.7)	4863 (20.7)	92 (5.4)	2911 (15.3)	3003 (14.4)
Skilled assistance	1087 (90.4) ***	12,483 (78.3)	13,570 (79.3)	902 (94.6) ***	10,136 (84.7)	11,038 (85.6)
Type of residence						
Urban	2383 (58.3)	15,420 (29.0)	17,803 (31.3)	2827 (46.7)	18,216 (29.1)	21,043 (30.9)
Rural	1682 (41.7) ***	39,791 (71.0)	41,473 (68.7)	4097 (53.3) ***	56,104 (70.9)	59,847 (69.1)
Parity of woman						
Under 5 children	1418 (37.6)	18,674 (34.3)	20,092(34.5)	2473 (40.8)	25,887 (37.1)	28,360 (37.5)
5 children and over	2647 (62.4)	36,537 (65.7)	39,184 (65.5)	4097 (59.2) ***	48,433 (62.9)	52,530 (62.5)
Wealth index						
Poorest	430 (8.9)	14,373 (21.2)	14,803 (20.3)	1025 (12.4)	21,991 (23.2)	23,016 (22.3)
Poorer	546 (11.7)	12,345 (22.3)	12,891 (21.5)	1155 (14.7)	19,716 (22.1)	20,871 (21.5)
Middle	556 (11.1)	12,154 (21.8)	12,710 (21.0)	1430 (19.5)	16,717 (21.1)	18,147 (20.9)
Richer	1071 (29.6)	9883 (19.3)	10,954 (20.1)	1711 (26.6)	10,764 (19.2)	12,475 (19.9)
Richest	1462 (38.7)	6456 (15.4) ***	7918 (17.1)	1249 (26.8) ***	5132 (14.4)	6381 (15.4)

Legend: (1) * < 0.05; (2) ** < 0.001; (3) *** < 0.0001.

**Table 5:** Difference-in-Differences estimates of the effect of modern contraceptive use on the probability of under-five mortalities, stratified by place of residence.

Variables	LPM [I]		LPM [II]		LPM [III]	
	β	IC95%	β	IC95%	β	IC95%
Treatment x year	0.015	[−0.005; 0.033]	−0.031 *	[−0.059; −0.002]	−0.010	[−0.026; 0.007]
Year	−0.045 ***	[−0.052; −0.037]	−0.035 ***	[−0.041; −0.029]	−0.038 ***	[−0.043; −0.033]
Treatment	−0.012	[−0.026; −0.002]	0.003	[−0.023; 0.030]	−0.005	[−0.019; 0.009]
Sex of child						
Female	−0.012	[−0.019; −0.005]	−0.017 ***	[−0.023; −0.011]	−0.016 ***	[−0.020; −0.011]
Age (year)	0.0002	[−0.0002; 0.0008]	−0.0001	[−0.0003; 0.0005]	−0.0002	[−0.0002; 0.0005]
Parity of mother						
More than 4	0.027 ***	[0.020; 0.035]	0.048 ***	[0.041; 0.055]	0.040 ***	[0.035; 0.045]
Wealth index						
Poorer	−0.007	[−0.027; 0.011]	−0.0007	[−0.007; 0.006]	−0.001	[−0.008; 0.006]
Middle	−0.012	[−0.027; −0.004]	−0.006	[−0.013; 0.017]	−0.007	[−0.013; 0.0002]
Richer	−0.013	[−0.027; 0.004]	−0.015 *	[−0.025; −0.005]	−0.014 **	[−0.022; −0.005]
Richest	−0.035 ***	[−0.08; −0.021]	−0.069 ***	[−0.090; −0.046]	−0.034 ***	[−0.042; −0.025]
Type of residence						
Rural	-	-	-	-	0.012 **	[0.006; 0.019]
Observations	38,436		99,837		138,273	
Stratification	Urban		Rural		All	

Legend: (1) * < 0.05; (2) ** < 0.001; (3) *** < 0.0001.

## Data Availability

The data supporting the findings of this study are publicly available and were obtained from the Demographic and Health Surveys (DHS) Program. The datasets used—specifically the DHS conducted in the Democratic Republic of the Congo in 2007, 2013–14, and 2023–24—can be accessed upon registration and request via the DHS Program website: https://dhsprogram.com/data/available-datasets.cfm/ (accessed on 15 July 2025). All data used were anonymized and are provided for research purposes only.

## Abbreviations

The following abbreviations are used in this manuscript:

ANC	Antenatal Care
AOR	Adjusted Odds Ratio
CI	Confidence Interval
CTMP	Comité technique multisectorielle de pilotage
DHS	Demographic and Health Survey
DiD	Difference-in-Differences
DRC	Democratic Republic of the Congo
FP	Family Planning
FP2020	Family Planning 2020 Initiative
FP2030	Family Planning 2030 Initiative
GLM	Generalized Linear Model
HRP	High-Risk Pregnancy
LPM	Linear Probability Model
MELOGIT	Multilevel Mixed Effects Logistic Regression
MMR	Maternal Mortality Ratio
MoPH	Ministry of Public Health
SDG	Sustainable Development Goal
SDM	Standard Days Method
TFR	Total Fertility Rate
U5MR	Under-Five Mortality Rate
UHC	Universal Health Coverage
WHO	World Health Organization
